# Hypoxia inducible factors regulate the transcription of the sprouty2 gene and expression of the sprouty2 protein

**DOI:** 10.1371/journal.pone.0171616

**Published:** 2017-02-14

**Authors:** Xianlong Gao, Kristin C. Hicks, Paul Neumann, Tarun B. Patel

**Affiliations:** 1 Department of Surgery, Loyola University Chicago, Chicago, Illinois, United States of America; 2 Department of Pharmaceutical Sciences, Albany College of Pharmacy and Health Sciences, Albany, New York, United States of America; Duke University, UNITED STATES

## Abstract

Receptor Tyrosine Kinase (RTK) signaling plays a major role in tumorigenesis and normal development. Sprouty2 (Spry2) attenuates RTK signaling and inhibits processes such as angiogenesis, cell proliferation, migration and survival, which are all upregulated in tumors. Indeed in cancers of the liver, lung, prostate and breast, Spry2 protein levels are markedly decreased correlating with poor patient prognosis and shorter survival. Thus, it is important to understand how expression of Spry2 is regulated. While prior studies have focused on the post-translation regulation of Spry2, very few studies have focused on the transcriptional regulation of *SPRY2 gene*. Here, we demonstrate that in the human hepatoma cell line, Hep3B, the transcription of *SPRY2* is inhibited by the transcription regulating hypoxia inducible factors (HIFs). HIFs are composed of an oxygen regulated alpha subunit (HIF1α or HIF2α) and a beta subunit (HIF1β). Intriguingly, silencing of HIF1α and HIF2α elevates *SPRY2* mRNA and protein levels suggesting HIFs reduce the transcription of the *SPRY2* promoter. *In silico* analysis identified ten hypoxia response elements (HREs) in the proximal promoter and first intron of *SPRY2*. Using chromatin immunoprecipitation (ChIP), we show that HIF1α/2α bind near the putative HREs in the proximal promoter and intron of *SPRY2*. Our studies demonstrated that not only is the *SPRY2* promoter methylated, but silencing HIF1α/2α reduced the methylation. ChIP assays also showed DNA methyltransferase1 (DNMT1) binding to the proximal promoter and first intron of *SPRY2* and silencing HIF1α/2α decreased this association. Additionally, silencing of DNMT1 mimicked the HIF1α/2α silencing-mediated increase in *SPRY2* mRNA and protein. While simultaneous silencing of HIF1α/2α and DNMT1 increased SPRY2 mRNA a little more, the increase was not additive suggesting a common mechanism by which DNMT1 and HIF1α/2α regulate *SPRY2* transcription. Together these data suggest that the transcription of *SPRY2* is inhibited by HIFs, in part, via DNMT1- mediated methylation.

## Introduction

Sprouty (Spry) proteins were first discovered in Drosophila melanogaster as inhibitors of fibroblast growth factor receptor-induced tracheal branching [1]. Subsequently, four mammalian isoforms of Sprouty (Spry1, Spry2, Spry3, and Spry4) were identified that are transcribed from four different genes. The different Spry isoforms have been shown to modulate the actions of receptor tyrosine kinases (RTKs); therefore, Spry proteins play a role in processes that require extensive RTK signaling such as organogenesis and tumorigenesis [2–5]. Specifically in development, Spry proteins have been shown to regulate the process of angiogenesis, patterning of the midbrain and anterior hindbrain, and development of the kidneys, lungs, limb buds, craniofacial features, and trunk [4,6–11]. After development Spry proteins continue to regulate angiogenesis [4,12–14], cell proliferation [15,16], migration [16–19] and survival [20,21]. Likewise, the role of Spry proteins, mainly Spry1 and Spry2, in cancer has also been investigated. Previous research has shown that the levels of Spry1 and Spry2 are decreased in cancers of the breast [22], lung [23], liver [24–28], and prostate [29–31] correlating to poor patient prognosis. Due to the important role Spry proteins play in development and tumorigenesis, it is crucial to understand how Spry levels are regulated.

We focused on Spry2, which is ubiquitously expressed and most studied among the Spry isoforms. Prior studies have concentrated on the regulation of the Spry2 protein through a variety of posttranslational modifications such as ubiquitylation or phosphorylation (reviewed [32,33]). However, early on Spry expression patterns during development were assessed and showed that the transcription of *SPRY* is upregulated by growth factors [34–37] elevating Spry protein levels in the centers of growth factor signaling (e.g. limb buds), thereby optimizing the ability of Spry proteins to act as negative feedback inhibitors of the enhanced RTK signaling in these areas. Additionally, while many other transcription factors have been predicted to bind to the *SPRY2* promoter, few have actually been shown to bind [38]. Ding et al. performed a functional analysis of the *SPRY2* promoter and identified that Ap2, Ets-GATA, and SP-1 bind to the *SPRY2* promoter enhancing its transcription [38]. However, the functional significance of the binding of these transcription factors to the *SPRY2* promoter remains unknown.

Because Spry2 levels are reduced in some forms of cancer [22–29,31], the regulation of Spry2 in cancer has been investigated. Most studies, however, have focused on the post-transcriptional regulation of Spry2 [39–43]. The few studies that have investigated transcriptional regulation of *SPRY2* promoter have shown that FOXO and beta-catenin bind to the *SPRY2* promoter and induce its transcription [44,45]. In terms of epigenetic modifications, the promoters of Spry4 and Spry2 have been shown to be hypermethylated in prostate cancer [46], but not breast cancer [22]. Two reports suggest that *SPRY2* promoter is hypermethylated in hepatocellular carcinomas [27,47], but another report suggests otherwise [25].

In both development and tumorigenesis, cells experience a hypoxic environment to which they adapt to by upregulating the transcription factors, hypoxia inducible factors (HIFs). HIFs are composed of an oxygen-regulated alpha subunit (HIF1α/HIF2α) and a beta subunit (HIF1β a.k.a. aryl hydrocarbon receptor nuclear translocator (ARNT)). Opposite to the actions of Spry2, HIFs promote proliferation, migration, and survival of cells by increasing the transcription of a number of genes that regulate these processes (reviewed in [48–50]).

Because Spry2 protein levels are decreased in hepatocellular carcinomas [24–28] and given the opposite actions of Spry2 and HIF1α/HIF2α on cell proliferation and migration [15,16,19,36,37,48–53], we performed an *in silico* analysis of the *SPRY2* promoter for hypoxia response elements (HRE) with the consensus sequence 5’-A/GCGTG-3’ and found 10 putative HREs; five in the proximal promoter and five in the first intron. Therefore, the purpose of this study was to determine whether HIF1α/HIF2α regulated the transcription of the *SPRY2* promoter. Herein, we demonstrate that, in the hepatoma cell line Hep3B, endogenous HIF1α and HIF2α decreased the mRNA levels of *SPRY2* with a concomitant decrease in the protein levels of Spry2. While the stability of the *SPRY2* mRNA wasn’t altered by HIF silencing, inhibiting DNA methylation with decitabine (DAC) abolished the increase in *SPRY2* mRNA when the expression of HIF1α/2α were silenced. Chromatin Immunoprecipitation (ChIP) assays revealed HIF1α/2α bind to regions in both the proximal promoter and first intron, each of which contains four and five HIF1α/HIF2α binding sites, respectively. Methylation of the proximal promoter of *SPRY2* was also observed and HIF1α/2α silencing decreased this methylation. Furthermore, ChIP assays revealed association of DNA methyltransferase 1 (DNMT1) with the proximal promoter and first intron of *SPRY2* and silencing of HIF1α/2α diminished this interaction. Finally, silencing of DNMT1 mimicked the actions of HIF1α/2α silencing in elevating *SPRY2* mRNA and protein levels. However, simultaneous silencing of DNMT1 and HIF1α/2α did not elevate *SPRY2* mRNA or protein levels additively suggesting that DNMT1 and HIF1α/2α work through a common mechanism. These data suggest that HIF1α/2α suppress *SPRY2* transcription, in part by increasing *SPRY2* promoter methylation by DNMT1.

## Results

### HIF1α and HIF2α decrease the mRNA and protein levels of Spry2

**Fig 1:**
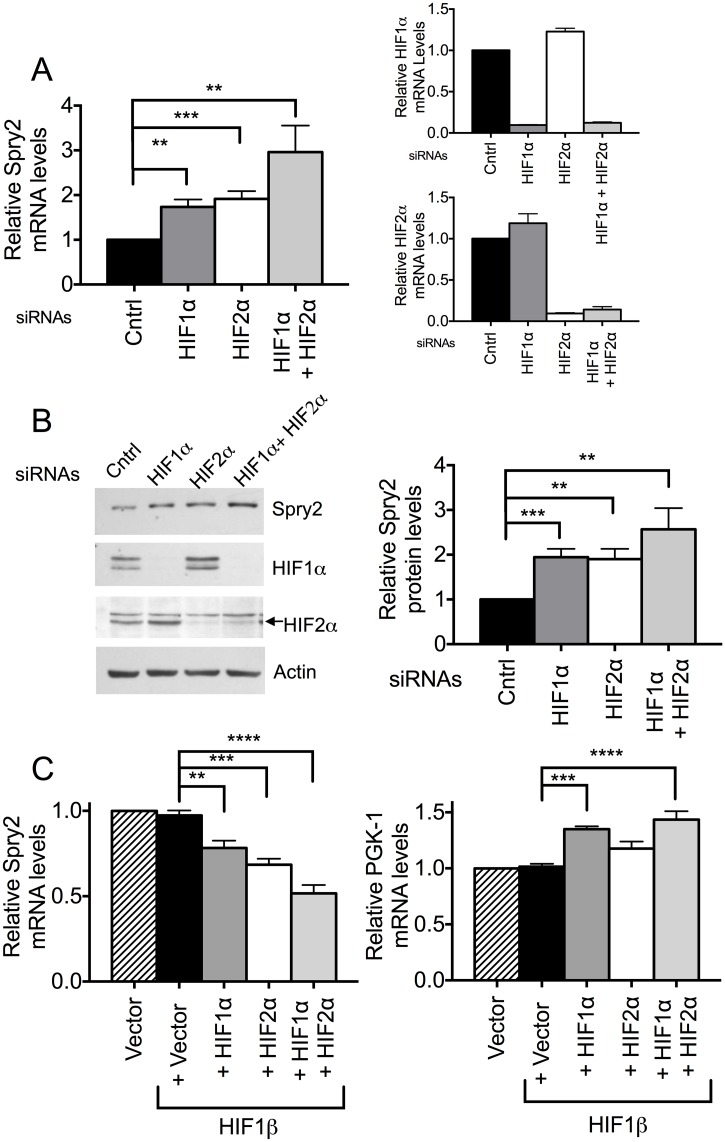
HIF1α and HIF2α regulate mRNA and protein levels of Spry2. (A) Cells transfected with siRNA against HIF1α, HIF2α or both isoforms were incubated under hypoxic conditions (3% O_2_) for 24 hours. RNA was isolated and mRNA levels of *HIF1α*, *HIF2α* (right panels) and *SPRY2* (left panel) were monitored by qRT-PCR with specific primers/probe and normalized with 18S rRNA. Cells transfected with mutant siRNA were used as control. Graphs are mean + SEM of 5 independent experiments. (B) Cells were treated same as in (A) except hypoxic incubation was for 32 hours. The protein levels of HIF1α, HIF2α and Spry2 were analyzed by Western blotting. Actin was used as loading control. Graph is mean + SEM from six independent experiments. (C) HEK293T cells transfected with vector alone or HIF1β along with vector, HIF1α, HIF2α, or both HIF1α and HIF2α were incubated under normoxic conditions for 40 hours after transfection. The mRNA amounts of *SPRY2* (left panel) or *PGK1* (right panel) were monitored by qRT- PCR and normalized with 18S rRNA. Graphs are mean + SEM from four independent experiments. Each group was compared with cells transfected with pcDNA3-HIF1β only. Statistical significance was assessed using unpaired Student t-tests (A & B) or one-way ANOVA with Dunnett’s multiple comparison test (C) **: *p*<0.01, ***: *p*<0.001, ****: *p*<0.0001.

To investigate if HIF1α and HIF2α regulated *SPRY2* mRNA and protein levels, we used siRNAs to silence the expression of endogenous HIF1α and HIF2α proteins in the hepatoma cell line Hep3B. While the silencing of HIF1α or HIF2α separately elevated *SPRY2* mRNA levels by about 100% each, silencing of both HIF1α and HIF2α together more profoundly (200%) elevated *SPRY2* mRNA levels (Fig 1A, left panel); the efficient silencing of both HIF1α and HIF2α by the siRNAs is also shown in Fig 1A (right panels). Consistent with the change in *SPRY2* mRNA levels, an increase in Spry2 protein levels was observed with HIF1α and HIF2α silencing. Here again, silencing both HIF1α and HIF2α had a larger effect on Spry2 protein levels, increasing them by about 150%, while silencing either HIF1α or HIF2α alone increased Spry2 protein to a lesser extent (~100%) (Fig 1B, right panel).

Conversely, in HEK293T cells, ectopic expression of HIF1α or HIF2α alone or both isoforms together decreased *SPRY2* mRNA levels. Once again, the expression of both HIF1α and HIF2α had a more profound effect on *SPRY2* mRNA levels by reducing them by about 50% while the expression of either HIF1α or HIF2α only reduced *SPRY2* mRNA by 20% and 30%, respectively (Fig 1C, left panel). Notably, we found that the co-expression of HIF1β, which dimerizes with HIF1α and HIF2α, is necessary to observe the effects of HIF1α or HIF2α overexpression, probably because endogenous HIF1β levels were not adequate to dimerize with the expressed HIFα subunits. However, HIF1β expression by itself does not alter *SPRY2* mRNA levels (Fig 1C, left panel). As a positive control to ensure that overexpressed HIF1α was modulating transcription appropriately, we monitored the mRNA levels of the HIF1α responsive gene phosphoglycerate kinase 1 (*PGK-1*) [54]. As expected, co-expressing HIF1α and HIF1β elevated *PGK-1* mRNA while expression of HIF1β alone or together with HIF2α had no effect (Fig 1C, right panel). Together, these data suggest that both HIF1α and HIF2α contribute to the decrease of the mRNA and protein levels of Spry2.

### HIF1α and HIF2α do not alter the stability of *SPRY2* mRNA, but HIF1α and HIF2α bind to the proximal promoter and intron of *SPRY2*

**Fig 2:**
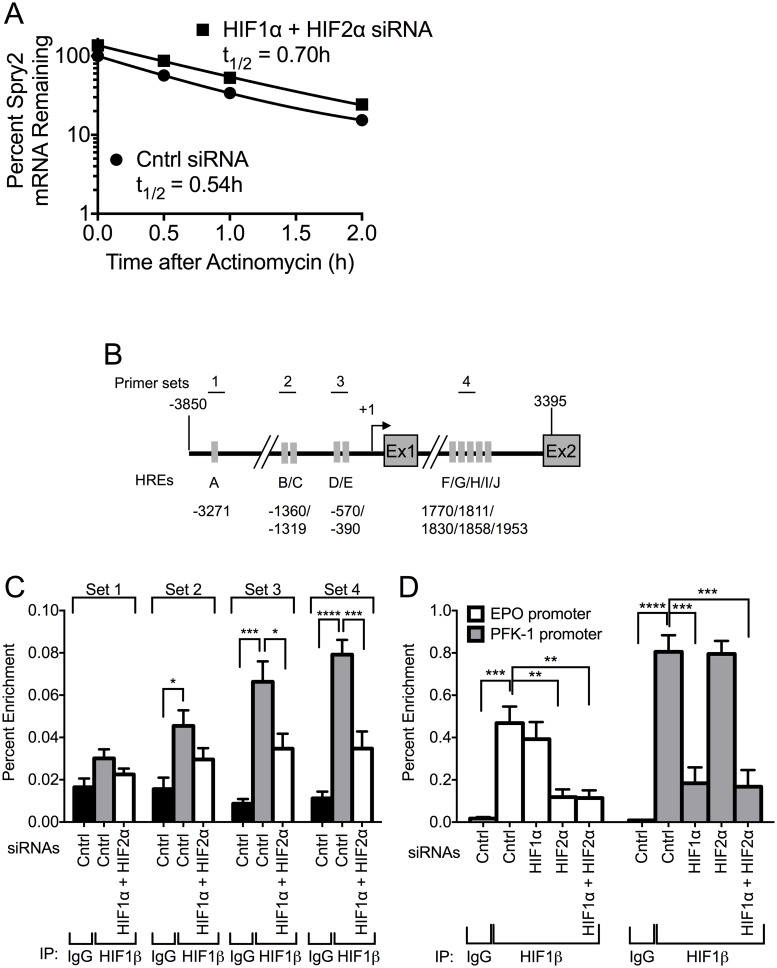
HIF1α and HIF2α do not regulate the stability of *SPRY2* mRNA, but they bind to the proximal promoter and intron of *SPRY2*. (A) Hep3B cells transfected with control or HIF1α/HIF2α siRNAs were incubated in hypoxia for 24 hours and then treated with actinomycin D (3 μg/mL). Total RNA was extracted at the indicated times and the mRNA levels of *SPRY2* were monitored using qRT-PCR. (B) Schematic of *SPRY2* from -3850 to 3395 encompassing the promoter, transcription start site (+1), exon 1 (Ex1), intron, and exon 2 (Ex2). Each grey rectangle labeled with a letter represents a putative HRE and the location of each HRE is labeled underneath. Each numbered line above shows the location of a primer pair designed to amplify a region of DNA with specific putative HREs in a ChIP. (C) Hep3B cells transfected with control or HIF1α and HIF2α siRNAs were incubated in hypoxia for 32 hours. Proteins, cross-linked to DNA, were immunoprecipitated with control rabbit IgG or HIF1β antibody. The DNA was sheared and the amounts of co-immunoprecipitated DNA were examined by qRT-PCR with the indicated primer sets. Graphs are the mean + SEM from five independent experiments. (D) Hep3B cells transfected with control, HIF1α, HIF2α, or HIF1α and HIF2α siRNAs were incubated in hypoxia for 32 hours. ChIP assays were performed as stated in (C) except primers were used that encompass the HREs located in the promoter of the HIF1α-responsive gene PFK-1 or the HIF2α-responsive gene *EPO*. Graph shows the mean + SEM from three independent experiments. Statistical significance was assessed using one-way ANOVA with Dunnett’s multiple comparison test (C & D) *: *p*<0.05, **: *p*<0.01, ***: *p*<0.001, ****: *p*<0.0001.

Since changes in mRNA levels may reflect either an alteration in the half-life of the mRNA and/or the rate of its transcription, we first determined whether silencing of HIF1α/HIF2α modulated the stability of *SPRY2* mRNA. For this purpose, Hep3B cells transfected with control or HIF1α and HIF2α siRNAs were treated with actinomycin D to inhibit RNA synthesis and the mRNA levels of *SPRY2* were then monitored over 2 hours by qRT-PCR. As shown in Fig 2A, during the 2 hours of actinomycin D treatment, *SPRY2* mRNA levels were reduced by approximately 80%. However, the rate of *SPRY2* mRNA degradation was not significantly different whether or not HIF1α and HIF2α were silenced (Fig 2A); the half-life of *SPRY2* mRNA was 0.54 versus 0.70 hours in control versus HIF1α and HIF2α siRNA transfected cells, respectively. Interestingly, over the entire time course the cells with HIF1α and HIF2α silenced had higher *SPRY2* mRNA levels, which is to be expected given the observation that *SPRY2* mRNA levels are elevated when HIF1α and HIF2α are silenced (Fig 1A).

Since these data suggest HIF1α and HIF2α do not alter the stability of *SPRY2* mRNA, we next investigated if HIF1α and HIF2α regulate the transcription of *SPRY2* mRNA. It is well established that both HIF1α and HIF2α bind to Hypoxia Response Elements (HREs) with the consensus sequence 5’-A/GCGTG-3’ in the promoter of the genes they regulate [55,56]. We performed an *in silico* analysis to determine if the *SPRY2* proximal promoter (-3850 to +1) or first intron (+1 to +3395) contained putative HREs. Indeed, we found 5 putative HREs in the proximal promoter located at positions -3271, -1360, -1319, -570, and -390 and 5 putative HREs in the first intron located at nucleotides 1770, 1811, 1830, 1858, and 1953 (Fig 2B). Furthermore, some of the HREs are conserved amongst primates as well as mice, rats, and rabbits (S1 Table).

To determine if HIF1α and HIF2α could bind any of these putative HREs, we performed a chromatin immunoprecipitation (ChIP) assay using a HIF1β antibody to immunoprecipitate both HIF1α and HIF2α bound DNA. We then used primers targeted against 4 different HRE containing areas of the proximal promoter and intron of *SPRY2* to quantify the amount of DNA that was immunoprecipitated by HIF1β. The location of the primers is shown in Fig 2B. Intriguingly, there was no significant enrichment of HIF1α/HIF2α/HIF1β on the HRE located at nt -3271 in the proximal promoter of *SPRY2* as shown by primer set 1 (Fig 2C). However, primer sets 2 and 3, targeting the four HREs closest to the transcription start site of *SPRY2*, and primer set 4, targeting the intron of *SPRY2*, showed significant enrichment of HIF1α/HIF2α/HIF1β. Furthermore, silencing of HIF1α and HIF2α significantly reduced the DNA enrichment in ChIP assays with primer sets for these sites (Fig 2C). As a positive control, by identical ChIP assays, we monitored the enrichment of the HREs located in the promoter of the HIF1α target gene phosphofructokinase (PFK) [57] and HIF2α target gene erythropoietin (*EPO*) [58,59]. As expected, the immunoprecipitation of HIF1β was greatly enriched with the DNA corresponding to HREs in the promoters of both PFK and *EPO* genes, and silencing of HIF1α or HIF2α greatly diminished the DNA enrichment from PFK and *EPO* promoters, respectively (Fig 2D). These latter findings authenticate that the combination of ChIP assays with HIF1β antibody and HIF1α/HIF2α silencing is a valid approach to study HREs on promoters. Together the data in Fig 2 suggest that HIF1α and HIF2α do not alter the stability of *SPRY2* mRNA, but both HIF1α and HIF2α bind to the proximal promoter and first intron of the *SPRY2* gene.

### HIF1α and HIF2α regulate *SPRY2* mRNA levels by modulating the methylation of the *SPRY2* promoter

The data in Figs 1 and 2 suggest that HIF1α/HIF2α bind to *SPRY2* promoter and first intron to repress the expression of Spry2. While studies have shown that hypoxia represses a set of genes, not many studies have shown that HIF1α or HIF2α specifically repress gene transcription. Furthermore, the precise mechanisms of repression are unknown or vary depending on the gene [60–64]. Methylation of promoters is well known to repress gene transcription [65] and hypoxia and HIF1α have been shown to modulate methylation of genes [66,67]. Therefore, we investigated whether the methylation status of the *SPRY2* promoter: (a) altered *SPRY2* mRNA levels, (b) modulated the ability of HIF1α/HIF2α silencing to alter *SPRY2* mRNA levels, and (c) was regulated by endogenous HIF1α/HIF2α.

**Fig 3:**
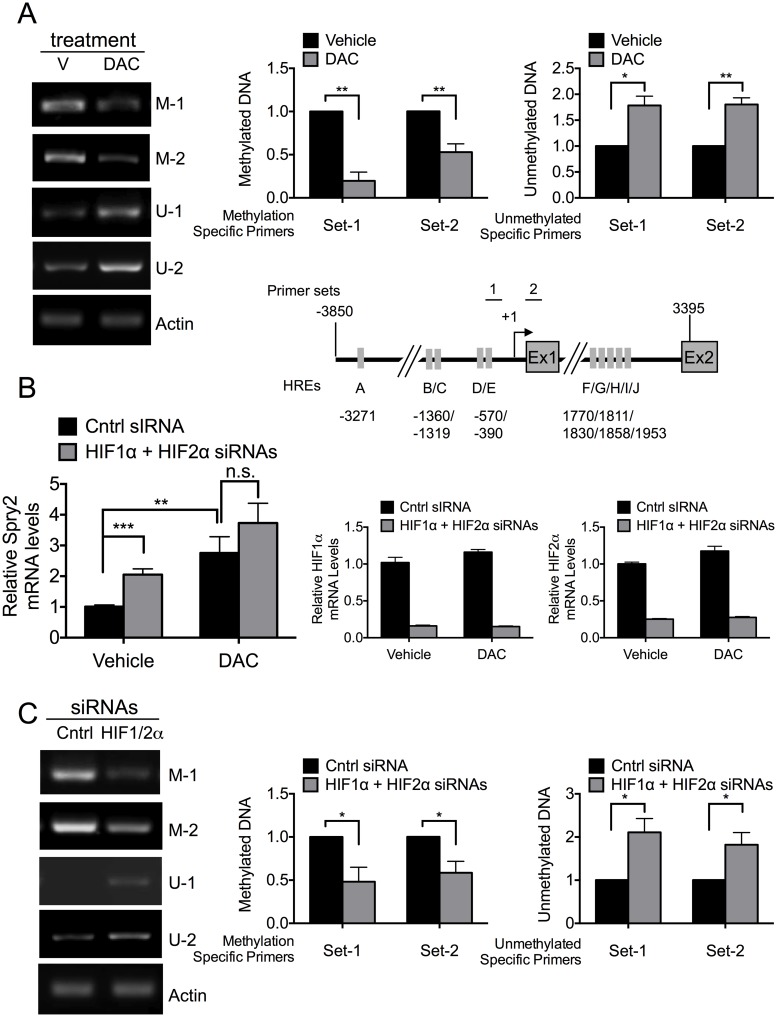
HIF1α and HIF2α repress *SPRY2* mRNA levels by enhancing the methylation of the *SPRY2* promoter. (A) *Upper panel*: Hep3B cells treated with vehicle (V) or decitabine (DAC) were incubated in hypoxia for 24 hours. DNA was extracted, bisulfite-converted, and the methylation status was assessed with methylation specific (M) and unmethylated specific (U) PCR primers. The amount of β-actin DNA was monitored to control for DNA amount loaded into each PCR. The amounts of methylated and unmethylated *SPRY2* promoter DNA were quantified by densitometry and normalized to β-actin. Graph shows the mean + SEM for three independent experiments. *Lower panel*: Schematic of *hSPRY2* promoter and gene showing the positions of PCR primers for both methylation specific and unmethylated specific PCRs. The arrow shows the transcription start site. Grey rectangles depict putative HREs. (B) Hep3B cells were treated with vehicle or decitabine (DAC, 5 μM), transfected with control or HIF1α and HIF2α siRNAs and incubated in hypoxia for 24 hours. RNA was isolated and the mRNA amounts of *SPRY2* (left panel), *HIF1α* and *HIF2α* (right panels) were monitored by qRT-PCR and normalized with 18S rRNA. Graphs show the mean + SEM from three independent experiments repeated in duplicate or triplicate. (C) Hep3B cells transfected with control or HIF1α and HIF2α siRNAs were incubated in hypoxia for 24 hours. The methylation status of the *SPRY2* promoter was analyzed as in (A). Graph shows the mean + SEM from five independent experiments. Statistical significance was assessed using unpaired student t-tests (A, B & C) *: *p*<0.05, **: *p*<0.01, ***: *p*<0.001, n.s: not significant.

By treating cells with decitabine (DAC), an inhibitor of DNA methyltransferases [68,69], we first determined whether the CpG islands in the proximal *SPRY2* promoter were methylated. Using bisulphite-treated genomic DNA and primers that specifically detect methylated (M-1, M-2) and unmethylated (U-1, U2) DNA corresponding to the regions 1 and 2 on the *SPRY2* promoter shown in Fig 3A inset (also see S2 Table), we determined the methylation status of the *SPRY2* promoter after treatment with DAC or its vehicle. As shown in Fig 3A, DAC treatment significantly decreased the methylation of the *SPRY2* promoter at primer sites 1 and 2 by 80% and 47%, respectively. Concomitantly, as expected, the amount of unmethylated promoter monitored by unmethylated DNA-specific primers for sites 1 and 2 was elevated by 78% and 80%, respectively. These findings demonstrate that the *SPRY2* promoter is methylated and DAC treatment effectively reduces its methylation.

Next, we determined whether treatment of cells with DAC altered *SPRY2* mRNA levels and the ability of HIF1α/HIF2α silencing to further modulate the amount of *SPRY2* mRNA. As shown in Fig 3B, DAC treatment of cells increased *SPRY2* mRNA levels by nearly 2-fold. However, while HIF1α/HIF2α silencing in vehicle treated cells (control) elevated *SPRY2* mRNA levels by ~100%, in the presence of DAC, silencing of HIF1α/HIF2α did not significantly increase *SPRY2* mRNA levels (Fig 3B); the efficient silencing of HIF1α and HIF2α is shown in the right panels of Fig 3B. These data in Fig 3A and 3B suggest that in Hep3B cells the reduction in methylation of the *SPRY2* promoter by DAC increases *SPRY2* mRNA levels and that DNA methylation plays a role in HIF1α/HIF2α- mediated decrease of *SPRY2* mRNA levels.

To directly assess whether silencing of HIF1α/HIF2α altered the methylation status of the *SPRY2* promoter, using primers corresponding to regions 1 and 2 on the *SPRY2* promoter that specifically recognize methylated (M-1, and M-2) vs. unmethylated DNA (U-1 and U-2), we determined the methylations status of the *SPRY2* proximal promoter with and without HIF1α/HIF2α silencing. As shown in Fig 3C, silencing of HIF1α and HIF2α decreased *SPRY2* promoter methylation detected by M-1 and M-2 primers by 52% and 42%, respectively, and increased the amounts of unmethylated *SPRY2* promoter monitored by U-1 and U-2 primers by 111% and 82%, respectively. Overall, these data (Fig 3) suggest that the *SPRY2* promoter is methylated in Hep3B cells, methylation of the promoter represses the expression of *SPRY2* mRNA, and endogenous HIF1α and HIF2α increase the methylation of the S*PRY2* promoter to decrease Spry2 mRNA levels.

### HIF1α and HIF2α regulate binding of DNMT1 to the *SPRY2* proximal promoter and intron and DNMT1 contributes toward HIF1α and HIF2α- mediated regulation of *SPRY2* mRNA

DNA methylation is a predominant epigenetic modification in mammals and is catalyzed by DNA methyltransferases (DNMTs), which are the enzymes that methylate the 5-position of cytosine in DNA primarily within a CpG dinucleotide [70]. There are four DNMT isoforms: DNMT1, DNMT3a, DNMT3b and DNMT3L. DNMT1 is the most abundant of these enzymes and is involved in maintaining methylation pattern by methylating newly replicated DNA [71,72]. DNMT3a and DNMT3b are considered *de novo* methyltransferases, since they add methyl groups to completely unmethylated DNA during development of an embryo [73]. DNMT3L does not possess any inherent enzymatic activity [74]. Intriguingly, hypermethylation of specific promoter regions has been implicated in promoting tumorigenesis (reviewed in [75]). One potential cause of this hypermethylation is the upregulation of DNMT’s, in particular DNMT1 [76–78]. In fact, previous studies showed that expression of DNMT1 in cultured cells increased CpG island methylation and resulted in cellular transformation [79,80]. With this in mind, we focused our studies on DNMT1.

**Fig 4:**
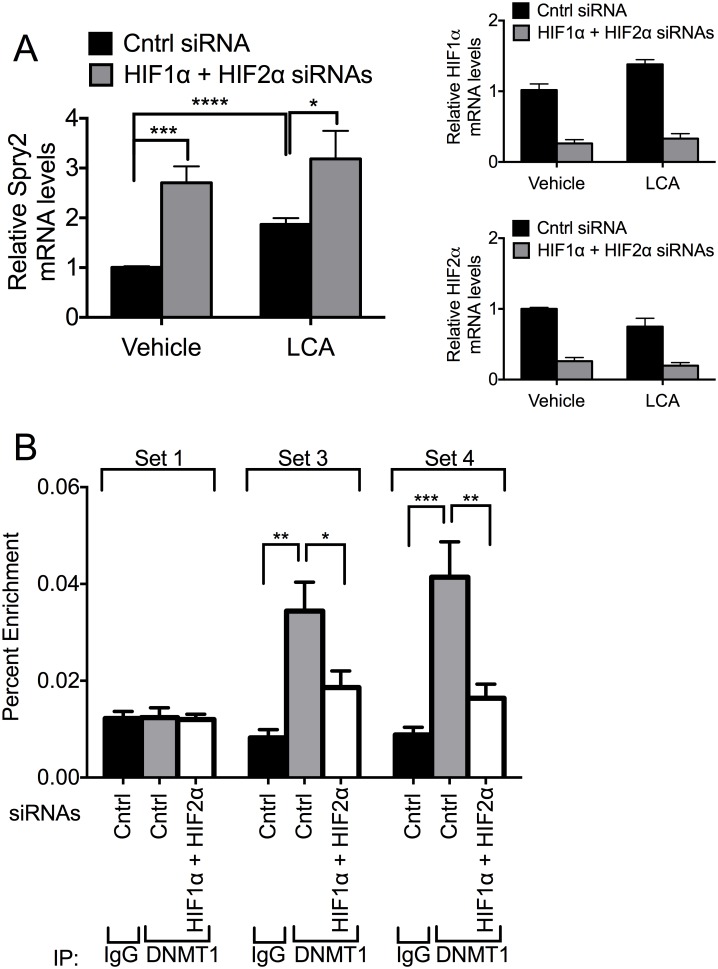
DNMT1 contributes toward the suppression of *SPRY2* mRNA expression by HIF1α and HIF2α. (A) Hep3B cells were treated with vehicle or laccaic acid A (LCA, 50 μg/mL), transfected with control or HIF1α and HIF2α siRNAs and incubated in hypoxia for 24 hours. RNA was isolated and mRNA amounts of *SPRY2* (left panel), *HIF1α*, and *HIF2α* (right panels) were quantified by qRT-PCR and normalized with 18S rRNA. Graphs show the mean + SEM from three independent experiments in duplicate. (B) Hep3B cells transfected with control or HIF1α and HIF2α siRNAs were incubated in hypoxia for 32 hours. Proteins, cross-linked to DNA, were immunoprecipitated with control mouse IgG or a DNMT1 antibody. The DNA was sheared and the amounts of co-immunoprecipitated DNA were examined by qRT-PCR with the indicated primer sets. Location of binding of primers is indicated in Fig 2B. Graphs show the mean + SEM from three independent experiments performed in singles or duplicates. Statistical significance was assessed using unpaired student t-tests (A) or one-way ANOVA with Dunnett’s multiple comparison test (B). *: *p*<0.05, **: *p*<0.01, ***: *p*<0.001, ****: *p*<0.0001.

Given our observations that HIF1α/HIF2α alter methylation of the *SPRY2* promoter (Fig 3C) and the recent findings demonstrating that laccaic acid (LCA) is a direct inhibitor of DNMT1 activity [81], we investigated whether inhibition of DNMT1 with LCA altered the ability of HIF1α and HIF2α to regulate *SPRY2* mRNA levels. Consistent with the data shown in Figs 1A and 3B, silencing HIF1α and HIF2α resulted in a 170% increase in *SPRY2* mRNA (Fig 4A). Importantly, treatment of cells with LCA, the DNMT1 inhibitor, also increased *SPRY2* mRNA levels by 87% and attenuated the increase in the levels of Spry2 mRNA observed with HIF1α and HIF2α silencing (170% in control versus 70% in LCA treated) (Fig 4A, left panel); the efficient silencing of HIF1α and HIF2α is shown in Fig 4A (right panels). These data suggest that DNMT1 regulates transcription of *SPRY2* mRNA and is partially involved in the ability of HIF1α and HIF2α to repress *SPRY2* mRNA levels.

To investigate if HIF1α and HIF2α altered the binding of DNMT1 to the *SPRY2* promoter, we performed ChIP assays with the DNMT1 antibody and monitored the amount of the proximal promoter and intron of *SPRY2* that immunoprecipitated with DNMT1 with and without silencing the expression of HIF1α/HIF2α. The location of the primer sets that bind to these regions is shown in Fig 2B. Similar to the ChIP assays shown in Fig 2B, DNMT1 immunoprecipitates were enriched with *SPRY2* gene regions corresponding to the promoter near the transcription start site (primer set 3 in Fig 2B) and the first intron of *SPRY2* gene (primer set 4 in Fig 2B) (Fig 4B). Additionally, silencing of HIF1α/HIF2α decreased the enrichment of DNA corresponding to these regions of the *SPRY2* gene in ChIP assays performed with the anti-DNMT1 antibody. These data suggest that DNMT1, either directly or indirectly, binds to the proximal promoter and first intron of the *SPRY2* gene and that HIF1α and HIF2α regulate the binding of DNMT1 to these regions.

One mechanism by which DNMT1 could be recruited to the *SPRY2* promoter and first intron is via interactions with HIF1α and/or HIF2α. However, despite numerous attempts using different conditions, we did not observe co-immunoprecipitation of HIF1α/HIF2α and DNMT1 (not shown) irrespective of which protein we immunoprecipitated. Thus, the precise mechanism by which HIF1α/HIF2α regulates binding of DNMT1 to the *SPRY2* promoter and first intron remains to be defined.

**Fig 5:**
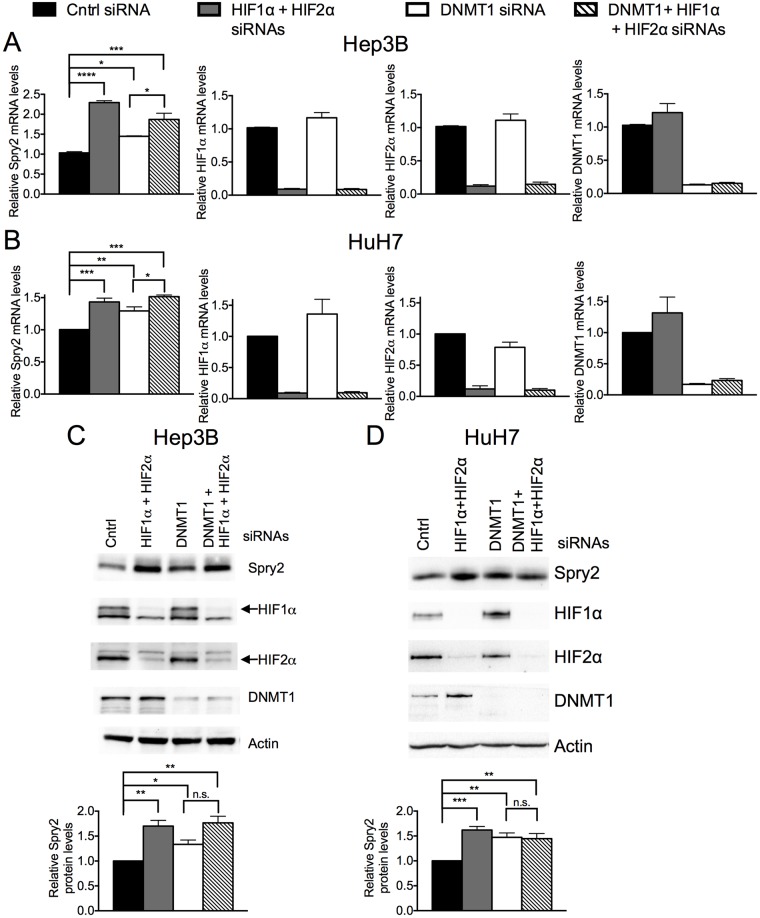
Silencing DNMT1 attenuates the increase in *SPRY2* mRNA and protein levels when HIF1α and HIF2α expression is silenced. (A) Hep3B and (B) HuH7 cells transfected with control, HIF1α and HIF2α, DNMT1, or DNMT1 and HIF1α and HIF2α siRNAs were incubated in hypoxia for 24 hours. RNA was isolated and mRNA amounts of *SPRY2*, *HIF1α*, *HIF2α*, and *DNMT1* were quantified by qRT-PCR and normalized with (A) 18S rRNA or (B) 18S rRNA and RPLP0. Graphs show the mean + SEM from three independent experiments in duplicate or triplicate. (C) Hep3B or (D) HuH7 cells were treated as in (A&B). The protein levels of DNMT1, HIF1α, HIF2α and Spry2 were analyzed by Western blotting. Actin was used as a loading control. Graph shows the mean + SEM from (C) three or (D) four independent experiments. Statistical significance was assessed using one-way ANOVA with Tukey’s multiple comparison test (A, B) or Sidak’s multiple comparison test (D) or unpaired student t-tests (B) *: *p*<0.05, **: *p*<0.01, ***: *p*<0.001, ****: *p*<0.0001, n.s: not significant. Key for bar graphs is at top.

To further elucidate the role of DNMT1 in the regulation of *SPRY2* mRNA levels by HIF1α/HIF2α, we monitored the levels of *SPRY2* mRNA in cells transfected with either control siRNA or siRNAs targeting HIF1α/HIF2α when DNMT1 expression was either silenced or not. In these studies, we included two hepatocellular carcinoma cell lines, HuH7 and Hep3B, to demonstrate the generality of the mechanisms investigated in this report. As shown in Fig 5A and as observed previously (Figs 1A, 2A, 3B & 4A), silencing of HIF1α/HIF2α in Hep3B cells increased *SPRY2* mRNA levels by 130%. The silencing of DNMT1 alone also increased *SPRY2* mRNA levels by ~50% (Fig 5A). The latter increase is consistent with the data in Fig 4A with LCA, suggesting that DNMT1, in part modulates *SPRY2* promoter activity. Of note, silencing of HIF1α/HIF2α had no effect on DNMT1 mRNA levels and vice versa (Fig 5A, right panels). Most interestingly, when DNMT1 and HIF1α/HIF2α expression was simultaneously silenced, *SPRY2* mRNA levels were not increased in an additive manner (Fig 5A). Similar results were observed in HuH7 cells with the exception that silencing of HIF1α/HIF2α elevated *SPRY2* mRNA levels by ~50% instead of the 100% increase observed in Hep3B cells (Fig 5B). Nevertheless, the other changes described for Hep3B cells with DNMT1 silencing alone and together with HIF1α/HIF2α silencing were also similar and statistically significant in HuH7 cells (Fig 5A & 5B). Consistent with changes in the *SPRY2* mRNA levels, silencing HIF1α/HIF2α or DNMT1 alone significantly elevated Spry2 protein levels in Hep3B cells (Fig 5C) and HuH7 cells (Fig 5D). However, as described above for *SPRY2* mRNA levels, simultaneous silencing of DNMT1 and HIF1α/HIF2α did not additively increase Spry2 protein levels (Fig 5D) suggesting that DNMT1 and HIF1α/HIF2α regulate Spry2 protein levels by a common mechanism. Together, the data in Figs 4 and 5 suggest that HIF1α and HIF2α regulate *SPRY2* mRNA and protein levels, in part, by regulating the binding of DNMT1 to the promoter and intron of *SPRY2*.

## Discussion

Ever since the discovery of the first *SPRY* gene in Drosophila, it has been clear that the Spry family of proteins, in a variety of species, play an important role in normal development of organs [33,82]. Because Spry proteins modulate the biological actions of growth factors that mediate their signaling via Receptor Tyrosine Kinases, a number of studies have examined the role that Spry proteins play in disease states associated with enhanced Receptor Tyrosine Kinase activities [32,33,82]. Essentially, these studies have shown that in certain disease states such as carcinomas of the breast, liver, lung and prostate, the levels of Spry proteins, especially Spry2, are decreased and probably contribute toward the pathogenesis of the disease [22–29,31]. Indeed, in hepatocellular carcinoma and breast cancer, a decrease in Spry2 levels has been correlated with poor prognosis and a decrease in patient survival [32,83]. For these reasons, it has been suggested to use Spry2 protein levels as a prognostic marker [24,32,83,84] and Spry proteins have been dubbed “tumor suppressors”. Thus, Spry proteins play an important role in normal development and in tumorigenesis.

During development and tumorigenesis, rapid cell proliferation that precedes angiogenesis exposes cells to hypoxia. The cells adapt to hypoxia by stabilizing the Hypoxia Inducible Factors, HIF1α and HIF2α, which are transcription factors that increase the expression of certain genes that promote cell survival and proliferation (reviewed in [48–50]). As such, in cancerous states, HIFs can be considered tumor promoters. Recently, we demonstrated that one of the mechanisms by which Spry2 exerts its “tumor suppressor” functions is by decreasing the stability of HIF1α and HIF2α with a corresponding decrease in their ability to alter transcription of the HIF1α and HIF2α target genes [85]. Herein, we asked the opposite question i.e. do HIF1α and HIF2α regulate Spry2 levels?

The evidence presented in this report demonstrates that endogenous HIF1α and HIF2α decrease the amounts of *SPRY2* mRNA and protein as silencing of endogenous HIF1α and HIF2α elevate both *SPRY2* transcript and protein levels. Conversely, ectopically expressed HIF1α and HIF2α decrease the amounts of *SPRY2* mRNA. The increase in *SPRY2* mRNA levels is not the result of changes in stability of the transcript, but for the following reasons, is a function of the change in transcription of the *SPRY2* gene. First, HIF1α/HIF2α bind to the proximal promoter and first intron of the *SPRY2* gene that contains a total of nine putative HRE consensus sequences (4 in proximal promoter and 5 in first intron). Second, HIF1α and HIF2α increase the methylation of the *SPRY2* promoter and repress *SPRY2* mRNA expression. Third, the inhibitor of DNMT1, LCA, and DNMT1-specific siRNA augment *SPRY2* mRNA and protein levels mimicking the effect of HIF1α/HIF2α silencing had to a lesser extent. As discussed later, simultaneous silencing HIF1α/HIF2α with DNMT1 resulted in a significant increase only in *SPRY2* transcript and not Spry2 protein levels. However, this increase in SPRY2 mRNA with DNMT1 and HIF1α/HIF2α silenced was not additive suggesting DNMT1 and HIF1α/HIF2α regulate SPRY2 through a similar mechanism.

Moreover, our studies also show that DNMT1 binds the *SPRY2* promoter and silencing of HIF1α/HIF2α decreases this association of DNMT1 with the *SPRY2* promoter. These findings suggest that HIF1α/HIF2α, in some manner, recruit DNMT1 to the *SPRY2* promoter to alter the methylation state of the promoter and, therefore, transcription of the *SPRY2* gene. One obvious mechanism would be the association between HIF1α/HIF2α and DNMT1 to recruit DNMT1 to the *SPRY2* promoter. However, despite several attempts using different immunoprecipitation conditions to elucidate interactions between HIF1α/HIF2α and DNMT1, we have been unable to show that these proteins reside in the same complex. It is possible that HIF1α/HIF2α alter the expression of some other protein that then permits the recruitment of DNMT1 to the *SPRY2* promoter.

Since HIF1α/HIF2α recruit DNMT1 to the *SPRY2* promoter and first intron, it would be expected that silencing of DNMT1 would increase SPRY2 mRNA to the same extent as silencing of HIF1α/HIF2α alone or together with DNMT1. However, in both Hep3B and HuH7 cells, the silencing of DNMT1 alone elevated SPRY2 mRNA levels to a significantly lesser extent than when HIF1α/HIF2α and DNMT1 were silenced simultaneously (Fig 5A and 5B). These data suggest that other DNMTs may also contribute toward HIF1α/HIF2α-mediated methylation of the *SPRY2* promoter. Given the large number of other members of the DNMT family and possible involvement of histone modifications, the identification of the other mechanisms (besides DNMT1) that may contribute toward HIF1α/HIF2α- mediated regulation of *SPRY2* promoter methylation should be the subject of future studies. Notably, the extent to which DNMT1 silencing alone and in combination with HIF1α/HIF2α elevated Spry2 protein is not significantly different (Fig 5C and 5D). However, this may be the result of the semi-quantitative nature of Western blot quantification as compared to the more rigorous quantitative analyses of mRNA levels by real time PCR.

In the light of this report and our recently published findings [85], one very important aspect of the regulatory interactions between HIF1α/HIF2α and Spry2 that emerges is that Spry2 regulates the stability of the HIF1α/HIF2α proteins and thereby attenuates their ability to alter transcription of the HIF1α/HIF2α-responsive genes, such as those regulating glucose uptake and glycolysis that play a critical role in survival of cells in hypoxia. Conversely, HIF1α/HIF2α by regulating the methylation status of the *SPRY2* promoter repress expression of *SPRY2* mRNA and protein. Hence, there is a reciprocal cross talk between the “tumor suppressor”, Spry2, and “tumor promoters”, HIF1α/HIF2α. However, the extent to which one dominates over the other may rely on the expression of other pertinent proteins that play a role e.g. pVHL in terms of HIF1α/HIF2α stability regulation by Spry2 [85] and forms of DNMT that facilitate HIF1α/HIF2α-mediated alterations in *SPRY2* promoter methylation. These elements may account for the differences in the extent to which SPRY2 mRNA is elevated upon silencing of HIF1α/HIF2α or DNMT1 in Hep3B and HuH7 cells (Fig 5A & 5B) Nevertheless, the cross-regulation between Spry2 and HIF1α/HIF2α would allow equilibrium to be reached so that one protein does not overly regulate the other to alter biological outcomes. This scenario would be beneficial in normal development and one could envisage this cross talk to regulate growth of tumors to some extent. In this context, Lee et al. [27] reported that in HuH7 and Hep3B cells cultured in normoxia the *SPRY2* gene is not methylated. This would be expected since in normoxia HIF1α/HIF2α levels are low and, therefore, would not facilitate the recruitment of DNMT1 to the *SPRY2* promoter. Extending this to tumors, it would be expected that in the hypoxic zones of tumors, elevated HIF1α/HIF2α protein levels would methylate the SPRY2 promoter and first intron to a greater extent than in normoxic areas of the tumors. Although the stability of the Spry2 protein is enhanced in hypoxia [39], over time the decreased transcription of the SPRY2 gene in hypoxic regions of tumors would be expected to decrease the protein levels of Spry2, diminish “tumor suppressor” actions of Spry2, and reduce the ability of Spry2 to oppose the “tumor promoting” actions of HIF1α/HIF2α. Hence, targeting HIF1α and HIF2α in tumors would not only suppress the “tumor promoting” actions of these transcription factors but by elevating *SPRY2* gene transcription, elevate Spry2 protein levels and, therefore, the tumor suppressing actions of Spry2.

Interestingly, the HREs in *SPRY2* promoter are conserved in other mammalian species (S1 Table) and the promoters and introns of other human *SPRY* genes (*SPRY1*, *SPRY3*, *and SPRY4*) also contain putative HREs (S1 Fig). Also, SPRY1 and *SPRY4* promoters have been reported to be methylated [86–88]. Likewise, a previous study showed that SPRY4 mRNA levels are increased in hypoxia [89], while conflicting studies, perhaps due to cell type, showed SPRY1 mRNA levels either increased or decreased by hypoxia [14,90]. In Hep3B cells, we observed that Spry1 protein levels were increased while Spry4 protein levels were decreased when HIF1α and HIF2α were silenced (data not shown); Spry3 levels were undetectable in Hep3B cells (not shown). Thus, it is tempting to speculate that *SPRY1* gene is also regulated by HIF1α/HIF2α via mechanisms described in this report for *SPRY2*. On the other hand, *SPRY4* may be regulated by HIF1α and HIF2α in an opposing manner to *SPRY2* by an as yet to be identified mechanism. *SPRY3* promoter does not contain CpG islands and is not methylated [91] and is probably not regulated by HIF1α/HIF2α via a methylation-dependent mechanism.

Overall, our findings described here unveil a new mechanism by which *SPRY2* gene expression is regulated by HIF1α/HIF2α. By binding to regions of the proximal promoter and first intron of *SPRY2*, HIF1α and HIF2α increase the methylation of the *SPRY2* promoter. We identified DNMT1 as a contributor toward this process as silencing or inhibiting DNMT1 attenuated HIF1α/HIF2α silencing mediated elevations in *SPRY2* mRNA and protein. These findings demonstrate that HIF1α/HIF2α, by repressing the expression of Spry2, can decrease the anti-tumorigenic actions of Spry2 protein.

## Experiment procedures

### Reagents and antibodies

Actinomycin D was purchased from Calbiochem, and decitabine or 5-aza-2’-deoxycytidine (also called dacogen, DAC) was from Cayman Chemical. Laccaic acid A (LCA) was obtained from TCI America.

All siRNAs and PCR primers, including general PCR primers, real time PCR primers and primers for methylation specific and non-methylation specific PCR, were synthesized by Integrated DNA Technologies Inc. The sequences of the primers are listed in S2 Table.

Antibodies used for Western blotting and chromatin immunoprecipitation were from the following companies: Sprouty2 (against N-terminus, Sigma), HIF1α (BD Transduction Laboratories), HIF2α (R&D Systems), HIF1β (Santa Cruz Biotechnology), and DNMT1 (AbCam).

### Plasmids

Human full-length HIF1α is PCR amplified with primers carrying HindIII and NotI sites from HIF1α cDNA clone (OriGene Technologies, Inc.) and inserted in pcDNA3 at HindIII and NotI sites. HIF2α is PCR amplified with primers harboring BamHI and NotI sites from pOTB7-HIF2α (Thermo Scientific.) and inserted in pcDNA3 at the corresponding sites. Plasmid pcDNA3-HIF1β was kindly provided by Dr. Guo-Qiang Chen (Shanghai Jiaotong University, China). Plasmid pGL2-Pfkfb3/-3566 was kindly provided by Dr. Ramon Bartrons, University of Barcelona.

### Cell culture, hypoxia and treatments

Hep3B and HuH7 cells were obtained from Dr. Basabi Rana, University of Illinois, Chicago. HEK293T were incubated in DMEM supplemented with 10% FBS, penicillin (100 units/mL), and streptomycin (100μg/mL). Hep3B cells were incubated in MEM supplemented with non-essential amino acids, sodium pyruvate and HEPES in addition to 10% FBS, penicillin (100 units/mL), and streptomycin (100μg/mL). HuH7 cells were incubated in DMEM F12 1:1 supplemented with HEPES, 10% FBS, penicillin (100 units/mL), and streptomycin (100 μg/mL). For normoxic conditions, cells were maintained at ambient O_2_ levels (21% O_2_) and 5% CO_2_ at 37°C. For hypoxic conditions, cells were maintained at 3% O_2_ and 5% CO_2_ in a Coy Hypoxic Chamber (Grass Lake, Michigan) at 37°C. All media used for hypoxia experiments were pre-equilibrated under hypoxic conditions overnight before use.

To examine whether HIFs alter *SPRY2* mRNA stability, Hep3B cells treated with 3μg/mL actinomycin D after siRNA transfection and 24 h hypoxic exposure were lysed at the indicated times with Trizol for RNA extraction. To investigate the involvement of DNA methylation in the regulation of *SPRY2* mRNA expression by HIFs, Hep3B cells were incubated with DNA methylation inhibitors, decitabine (DAC) at 5μM or laccaic acid A (LCA) at 50 μg/mL, for 24 h the day after cells were plated. Subsequently, cells were transfected with siRNAs in fresh medium containing DAC or LCA and then maintained under hypoxia for another 24 h before use.

### Overexpression of HIF1α and HIF2α

HEK293T cells were seeded in 3.5-cm dishes at 2 x 10^5^ /dish and transfected next day with 250 ng each of pcDNA3-HIF1β, pcDNA3-HIF1α and/or pcDNA3-HIF2α as indicated using Transit2020 transfection reagent (Mirus) following the manufacturer’s instructions. The total amount of plasmids transfected into each dish was kept the same by adding empty vector pcDNA3. Cells were incubated under normoxic condition for 40 h after transfection before RNA extraction.

#### Silencing with siRNAs

Hep3B cells were plated in 3.5-cm dishes at 3 x 10^5^ /dish or HuH7 cells were plated in 3.5-cm dishes at 2 x 10^5^/dish. Next day, cells were transfected with mutant siRNA or siRNAs against HIF1α, HIF2α or both at 20 nM each or for the experiments in Fig 5, cells were transfected with mutant, HIF1α and HIF2α, DNMT1 alone, or HIF1α and HIF2α and DNMT1 siRNAs (20nM each) with TKO transfection reagent (Mirus). After overnight transfection, cells were incubated in the hypoxic chamber for 24 h (for mRNA detection) or 32 h (for Western blotting). The sequences of siRNAs are: mutant siRNA, sense 5'-GUC AGC AGA ACA AAA GUA GTT-3' and antisense 5'-CUA CUU UUG GUU CUG CUG ACT T-3'; HIF1α, sense 5’-GAA GGA ACC UGA UGC UUU AAC UUT G-3’ and antisense 5’-CAA AGU UAA AGC AUC AGG UUC CUU CUU-3’; HIF2α, sense 5'-GCU GGA GUA UGA AGA GCA AGC CUT C-3' and antisense 5'-GAA GGC UUG CUC UUC AUA CUC CAG CUG-3'.

### RNA isolation and real time PCR

Total RNA was isolated with Trizol reagent following the manufacturer’s protocol (Invitrogen). The extracted total RNA (500 ng) was then converted to cDNA with SuperScript VILO cDNA synthesis kit (Invitrogen) according to manufacturer’s instructions. To detect mRNA amounts for *HIF1α*, *HIF2α*, *SPRY2* and *PGK1*, real time PCR was performed with specific primers and probes (S2 Table) and FastStart Universal Probe Master Mix (Roche Life Science) using the CFX96 real-time PCR detection system (Bio-Rad). PCR conditions were optimized for the primers/probe for each gene. The mRNA amounts of each gene were normalized with 18S rRNA.

### Chromatin Immunoprecipitation (ChIP)

Hep3B cells were plated at 5 x 10^5^ /dish in 6-cm dishes. Next day, cells were transfected with siRNAs as stated above. An extra dish of cells were transfected in parallel with siRNA and trypsinized for cell counting before use. After a 32 h hypoxic incubation (3% O_2_), cells were crosslinked with 1% formaldehyde for 10 min at room temperature. The crosslinking reactions were terminated by incubating in 0.125 M glycine for 5 min at room temp. The cells were washed twice with cold PBS, scraped into cold PBS containing protease inhibitors (1 μg/mL aprotinin and 1 μg/mL pepstatin, 2 μg/mL leupeptin and 1 mM phenylmethylsulfonyl fluoride) and pelleted by centrifugation at 2000 rpm for 5 min. The cell pellet was resuspended in SDS lysis buffer (1% SDS, 10 mM EDTA, 50 mM Tris-HCl, pH 8.0) containing the above protease inhibitors (200 ml/1 x 10^6^) and incubated for 10 min on ice. The cell lysate was sonicated to shear DNA followed by centrifugation to remove pellet. The supernatant was diluted 10 times with dilution buffer (0.01% SDS, 1.1% Triton X-100, 1.2 mM EDTA, 16.7 mM Tris-HCl, pH 8.1, 167 mM NaCl plus the above protease inhibitors) and pre-cleared with salmon sperm DNA/protein G beads. The cleared supernatant (from 1 x 10^6^ cells) was incubated with 2.5μg anti-HIF1β antibody or rabbit IgG overnight at 4°C and immunoprecipitated with 25 μL protein G agarose beads the next day. For ChIP with anti-DNMT1 antibody, 1.5 x 10^6^ cells were used per ChIP and mouse IgG was used as control antibody. The immunoprecipitates were washed sequentially with the following buffers: low salt buffer (0.1% SDS, 1% Triton X-100, 2 mM EDTA, 20 mM Tris-HCl pH 8.0, 150 mM NaCl), high salt buffer (0.1% SDS, 1% Triton X-100, 2 mM EDTA, 20 mM Tris-HCl pH 8.0, 500 mM NaCl), LiCl wash buffer (0.25 M LiCl, 1% IGEPAL-CA630, 1% Sodium deoxycholate, 1 mM EDTA, 10 mM Tris-HCl pH 8.0), and TE (10 mM Tris-HCl, 1 mM EDTA). DNA was then eluted with elution buffer (1% SDS, 0.1 M NaHCO_3_) and the crosslinking was reversed by adding 20 μl 5 M NaCl and incubated at 65°C overnight. After purification with PCR purification kit (Qiagen), the amount of DNA that was immunoprecipitated with a specific protein was quantified by real time PCR using the indicated primers (locations are shown in Figs) and SYBR master mix (Roche Life Sciences). The results are presented as % enrichment (% of the input DNA was immunoprecipitated with the indicated antibody).

### Methylation-specific PCR

Genomic DNA was extracted from Hep3B cells using the DNeasy tissue extraction kit (Qiagen) following the manufacturer’s instructions. Subsequently, 0.5–1.0 μg of DNA from each sample was used for bisulphite conversion using the EpiTect fast bisulfite conversion kit (Qiagen). The converted DNA was then purified with the same kit from Qiagen. DNA methylation status of the *SPRY2* gene was examined by PCR employing two sets of primers that match the same sites with one specific for methylated (M) and the other for unmethylated (U) sequences. The sequences of primers are listed in S2 Table. Two rounds of PCR amplification were performed to detect the methylation status using FastStart PCR master kit (Roche Life sciences). The first round PCR amplification conditions used were one cycle of 95°C for 4 min followed by 25 cycles of 95°C for 30 s, 55°C for 30 s, 72°C for 60 s. The resultant PCR product was diluted 20 times and used as template for the second round PCR amplification, which employed the nested forward primers (n) and the same reverse primers as in the first round PCR. The PCR conditions used were one cycle of 95°C for 4 min followed by 35 cycles of 95°C for 30 s, 60°C for 30 s, 72°C for 60 s. The PCR products were loaded into agarose gels and the DNA methylation status was quantified by densitometry of the bands and normalized with beta-actin.

### Statistical analysis

One-way ANOVA was employed for multiple-group comparisons using GraphPad 6 software. For two-group comparison, Student’s t test was performed.

## Supporting information

S1 Fig Schematic of promoters for *SPRY1*, *SPRY3*, and *SPRY4*. Schematic of *SPRY1*, *SPRY3*, and *SPRY4* from -4000 to the end of the coding sequence encompassing the promoter, transcription start site (+1), exon 1 (Ex1), intron, and exon 2 (Ex2) as well as intron 2 and exon 3 (Ex3) for *SPRY1*. Each colored rectangle labeled with a letter represents a putative HRE and the location of each HRE is labeled underneath.
(TIFF)

S1 Table Conservation of putative HREs from *SPRY2* promoter in other mammals. The table above indicates with an “X” if the putative HRE from the human *SPRY2* promoter aligns with an HRE sequence in chimps, mice, rats, dogs, or dolphins.
(DOCX)

S2 Table Primer sequences 5’ to 3’. The sequences for the primers and probes used in real time PCR are listed. The primers for chIP and methylation specific PCR’s are listed. For methylation-specific primers, “M” designates a primer set that amplifies methylated *SPRY2* promoter and “U’ designates a primer set that amplifies *SPRY2* promoter that is not methylated. “F-n” indicates the nested forward primer that was used in the subsequent PCR following the first PCR with the forward and reserve primers listed as described under “Methylation-specific PCR” in the *Experimental Procedures*.
(DOCX)
