# Supplementary figures and images for: Hypoxia inducible factors regulate the transcription of the sprouty2 gene and expression of the sprouty2 protein
Source: PLoS One. 2017 Feb 14;12(2):e0171616. doi: 10.1371/journal.pone.0171616 (PMC5308774; doi:10.1371/journal.pone.0171616)

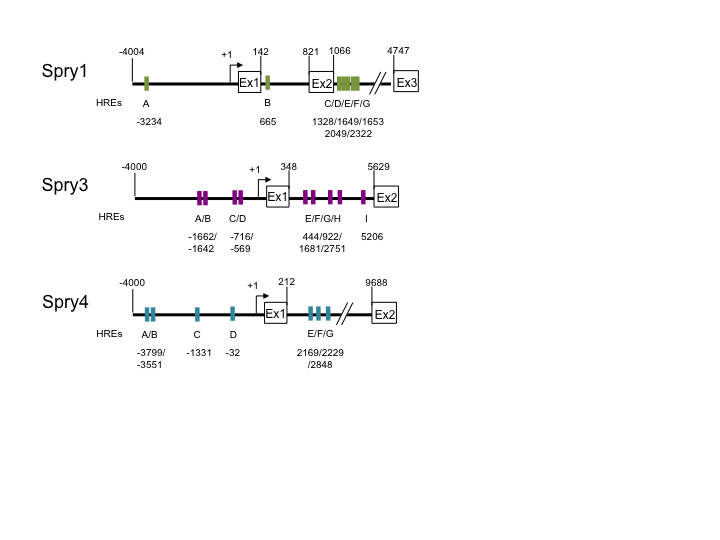

Supplement: S1 Fig — Schematic of SPRY1, SPRY3, and SPRY4 from -4000 to the end of the coding sequence encompassing the promoter, transcription start site (+1), exon 1 (Ex1), intron, and exon 2 (Ex2) as well as intron 2 and exon 3 (Ex3) for SPRY1. Each colored rectangle labeled with a letter represents a putative HRE and the location of each HRE is labeled underneath. (TIFF) [file pone.0171616.s001.tiff]

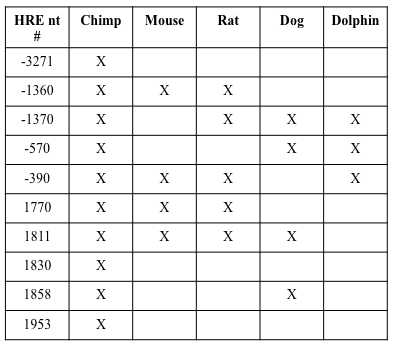

Supplement: S1 Table — The table above indicates with an “X” if the putative HRE from the human SPRY2 promoter aligns with an HRE sequence in chimps, mice, rats, dogs, or dolphins. (DOCX) [file pone.0171616.s002.docx]
