# Supplementary material for: Hypoxia inducible factors regulate the transcription of the sprouty2 gene and expression of the sprouty2 protein
Source: PLoS One. 2017 Feb 14;12(2):e0171616. doi: 10.1371/journal.pone.0171616 (PMC5308774; doi:10.1371/journal.pone.0171616)
Supplement: S2 Table — The sequences for the primers and probes used in real time PCR are listed. The primers for chIP and methylation specific PCR’s are listed. For methylation-specific primers, “M” designates a primer set that amplifies methylated SPRY2 promoter and “U’ designates a primer set that amplifies SPRY2 promoter that is not methylated. “F-n” indicates the nested forward primer that was used in the subsequent PCR following the first PCR with the forward and reserve primers listed as described under “Methylation-specific PCR” in the Experimental Procedures. (DOCX) [file pone.0171616.s003.docx]

| Real time PCR Primers and Probes | |
| --- | --- |
| **HIF1α** | F: AACATAAAGTCTGCAACATGGAAG  R: TTTGATGGGTGAGGAATGGG  Probe: TACGTGAATGTGGCCTGTGCAGT |
| **HIF2α** | F: CCCATGTCTCCACCTTCAAG  R: GGCTTGCTCTTCATACTCCAG  Probe: CGAGCCCCAAAACCCTTTGCAG |
| **Spry2** | F: TGCTCGGAAGTTGGTCTAAAG  R: CACATCTGAACTCCGTGATCG  Probe: TTGCACCAACCCCTCTCCCTT |
| **RN18S1** | F: GAGACTCTGGCATGCTAACTAG  R: GGACATCTAAGGGCATCACAG  Probe: TGCTCAATCTCGGGTGGCTGAA |
| **PGK1** | F: GCTTCTGGGAACAAGGTTAAAG  R: CTGTGGCAGATTGACTCCTAC  Probe: TCAATGATGCTTTTGGCACTGCTCAC |
| **DNMT1** | F: CCAGAGAACGAGTTGCTAGAC  R: CAGTTTCTGTTTGGGTGTTGG |
| **RPLP0** | F: GCAGCATCTACAACCCTGAA  R: CAGACAGACACTGGCAACAT |
| ChIP PCR Primers | |
| **Spry2 Set1** | F: CAGAGATGACAGATCCCAAGTG  R: CCTCCCAAAGTGCTGAGATT |
| **Spry2 Set2** | F: TGTAAGTCGCTCGACAGAATG  R: GTCCTAGCTGCCGTTCATTTA |
| **Spry2 Set3** | F: GGCAAGGAGAAAGTTCCCTAAG  R: GCACTGCAGGGTCAGTTTAT |
| **Spry2 Set4** | F: AGATAGGCGTGTGCAGATTAC  R: CAGGACGCACAAGTCCAA |
| **PFK1** | F: GACGTGAGCGACGTGTG  R: GGCTGTGATTGGAGGGAAG |
| **EPO** | F: CTGTTTTCTGGGAACCTCCA  R: GGAGCCACCTTATTGACCAG |
| Spry2 Methylation-specific PCR primers | |
| **M1** | F: TTTGTAGTGTTTAGTTCGGTTTCG  R: CAATAAATAACGTCATATAAATCCG  F-n: GGGCGGTAGGATCGGTTTGGGGAC |
| **U1** | F: TTTGTAGTGTTTAGTTTGGTTTTG  R: CAATAAATAACATCATATAAATCCA  F-n: GGGTGGTAGGATTGGTTTGGGATG |
| **M2** | F: TTTCGCGTCGTTTTCGTTACGACG  R: CCTTAAATTCTCTTCTTTCTACG  F-n: GGTTTTAGGTTTTTCGTAAGGTC |
| **U2** | F: TTTTGTGTTGTTTTTGTTATGATG  R: CCT TAAATTCTCTTCTTTCTACA  F-n: GGTTTTAGGTTTTTTGTAAGGTTG |
| **β-Actin** | F: TTTTATTTAGAGTGTAGGTGTGTGGAGATTTT  R: CAAAAACAAAAACCTAACCCCTAAACCT |
